# Retraction: Silencing of APE1 Enhances Sensitivity of Human Hepatocellular Carcinoma Cells to Radiotherapy In Vitro and in a Xenograft Model

**DOI:** 10.1371/journal.pone.0292822

**Published:** 2023-10-09

**Authors:** The PLOS ONE

Following the publication of this article [1], concerns were raised regarding Figs 4,8 and 9 Specifically,

- The P21 western blot panel in Fig 4A appears to have no detectable signal or background, nor does the panel present a positive control to confirm a successful blot.
- The Ad5/F35-siAPE1 panel in Fig 8A appears to partially overlap with the Ad5/F35-siAPE1+IR panel in Fig 8a and the Ad5/F35-siAPE1+IR panel in Fig 9a despite representing different experimental conditions.
- The Ad5/F35-siAPE1 panel in Fig 9A appears to partially overlap with the Ad5/F35-EGFP+IR panel when rotated despite representing different experimental conditions.

The authors did not provide a response to the concern regarding the P21 western blot in Fig 4A nor was any underlying data provided to resolve this issue.

The authors stated that the concerns related to Figs 8 and 9 were unintentional errors made during preparation of the figures but stated they were unable to source the original underlying data for both figures. The PLOS Editors remain concerned about the reliability of these figures.

In light of the concerns affecting multiple figure panels that question the reliability and validity of these data, the *PLOS ONE* Editors retract this article.

All authors either did not respond directly to the final editorial decision or could not be reached.
